# 3,3′-Diindolylmethane ameliorates renal fibrosis through the inhibition of renal fibroblast activation *in vivo* and *in vitro*

**DOI:** 10.1080/0886022X.2018.1490322

**Published:** 2018-08-12

**Authors:** Zun-En Xia, Juan-Li Xi, Lei Shi

**Affiliations:** a Department of Clinical Laboratory, Renmin Hospital of Wuhan University, Wuhan, China;; b Department of Gastroenterology, Wuhan Third Hospital, Wuhan, China;; c Department of Oncology, Renmin Hospital of Wuhan University, Wuhan, China

**Keywords:** 3,3′-Diindolylmethane, renal fibrosis, myofibroblast, TGF-β/Smad3, Smad7

## Abstract

3,3′-Diindolylmethane (DIM), a natural acid condensation extracted from cruciferous plants, exhibits anti-fibrotic effects in hepatic and cardiac fibrosis models. The effects of DIM on renal fibrosis, however, are unclear. This study aimed to explore the protective effects of DIM on renal fibrosis. Unilateral ureteral obstruction (UUO) and transforming growth factor (TGF)-β1-stimulated normal rat kidney (NRK)-49F fibroblast cell mouse models were established. The models were then treated with DIM for the assessment of its anti-fibrotic effects and mechanisms. Results of HE and Masson staining showed that DIM reduced kidney injury and production of interstitial collagens fibrosis. CTS also inhibited expression of fibronectin, collagen-1 but retain E-cadherin in the UUO model. Furthermore, DIM suppressed local fibroblast activation, as evidenced by the suppressed expression of the myofibroblast markers α-SMA and vimentin *in vivo* and *in vitro*. In addition, DIM significantly inhibited the TGF-β1-induced proliferation of NRK49F cells in a time- and dose-dependent manner. DIM decreased Smad2/3 phosphorylation but increased Smad7 expression. Results suggested that DIM inhibits TGF-β/Smad2/3 signaling to attenuate renal interstitial fibrosis via inhibiting local fibroblast activation. This mechanism is likely related to Smad7 induction.

## Introduction

The incidence of chronic kidney disease (CKD) has considerably increased over the past years. Thus, CKD has become a worldwide health problem that presents tremendous socioeconomic challenges. Renal interstitial fibrosis is a common characteristic of CKD and is observed in a variety of kidney diseases that causes renal failure [1]. Excessive interstitial matrix accumulation, tubular atrophy and dilation, inflammatory cell infiltration and myofibroblast activation are the primary pathological changes associated with renal interstitial fibrosis. Among these characteristic changes in fibrotic kidneys, the most crucial is the deposition of the interstitial extracellular matrix (ECM), which is mainly produced by myofibroblasts [2]. Mounting evidence demonstrates that proliferation and differentiation of interstitial fibroblasts are the predominant sources of myofibroblasts [3]. Hence, the prevention of interstitial fibroblast proliferation and the subsequent attenuation of fibroblast differentiation into myofibroblasts is a potential approach to the treatment of renal interstitial fibrosis [4].

Transforming growth factor-β (TGF-β) is the most important pro-fibrotic cytokine that induces the activation of renal fibroblasts [5]. During renal fibrosis, TGF-β1 exerts its biological activities via Smad-dependent signaling pathways. The activated TGF-β1 binds to its receptor II (TβRII), which recruits TGF-β receptor type-I (TβRI) kinases. Then, TβRI phosphorylates downstream Smad2 and Smad3. Subsequently, the phosphorylated Smad2/Smad3 form a complex by binding to Smad4. The complex can translocate into the nucleus to regulate the transcription of target genes [6]. Smad7 exerts protective effects during the progression of kidney fibrosis. Smad7 can be induced by TGF-β1 in a Smad3-dependent manner and competes with R-Smads for binding to activated receptors to exert its inhibitory effect on TGF-β/Smad signaling [7]. Thus, rebalancing the TGF-β/Smad signaling pathway through the upregulation of Smad7 and the suppression of Smad2/3 activation are prospective treatment options for renal fibrosis.

3,3′-Diindolylmethane (DIM), a natural compound derived from the acid-catalyzed self-condensation of indole-3-carbinol, is found in cruciferous vegetables, including turnips, broccoli, kale, cauliflower and cabbage [8]. Previous studies have indicated that DIM has numerous pharmacological effects, including anti-cancer [9], anti-inflammatory [10], and anti-angiogenic [11] effects. Recently, DIM has been reported to exert anti-hepatic and anti-cardiac fibrosis effects. For example, Lei Dong et al. reported that the DIM-inhibited TGF-β signaling pathway is a potential treatment strategy for hepatic fibrosis, and this pathway is involved in the activation of hepatic stellate cells by decreasing miR-21 expression [12]. Tang et al. reported that DIM improves cardiac hypertrophy and fibrosis in a cardiac hypertrophy mouse model through AMPKa2 activation [13]. Additionally, DIM decreases the TGF-β1-induced differentiation of cardiac myofibroblasts through the downregulation of AKT/GSK-3β signaling pathways [14]. Based on these findings, we hypothesize that DIM may inhibit myofibroblast differentiation to ameliorate renal fibrosis.

## Materials and methods

### Animal model

A total of 30 C57BL/6J mice (8-week old, 20–25 g) were obtained from Beijing HFK Bioscience Co., Ltd. (Beijing, China). The mice were housed in a temperature-controlled (21 ± 2 °C) animal center at the RenMin Hospital of Wuhan University. The animal studies were approved by the Institutional Animal Care and Use Committee of Wuhan University, China. The UUO model was performed by ligating the left ureter for 7 days as previously described [15]. Sham operation was conducted for all procedures, except for ureteral ligation. All mice were randomly assigned into three groups (*n* = 10): the sham, UUO plus vehicle (UUO + Vehicle) and UUO plus 100 mg/kg/day body weight DIM (Shanghai Medical Technology Development Co., Ltd., Harmony, China) groups. DIM dosage was selected based on our pilot test that aimed to improve the pathology staining of the UUO model. The mice received normal feed or feed containing 0.05% DIM (100 mg/kg/day). DIM was administered to mice four weeks prior to surgery. DIM administration continued until the experiment was terminated. The mice were sacrificed 7 days after surgery, and their kidneys were collected.

### Histological and immunohistochemical studies

Paraffin-embedded kidney tissues were sectioned to a thickness of 4 µm. Then the sections were dewaxed and hydrated in a graded ethanol series. Tubular injury after UUO was evaluated by hematoxylin and eosin (HE) staining in accordance with the manufacturer’s protocol (Sigma-Aldrich, St. Louis, MO, USA). Interstitial injury was scored in accordance with Dr. Tun-Jun Tsai’s methods as previously reported [16]. The score was defined as the quantity of necrotic tubule: 0, normal tubules; 1, rare single necrotic tubule; 2, several clusters of necrotic tubules; and 3, massive necrosis. Fibrotic cortical interstitial areas were detected by Masson trichrome staining. UltraVision Quanto Detection System HRP DAB kit (Thermo Fisher Scientific, Waltham, MA, USA) was used for immunohistological staining in accordance with the manufacturer’s instructions. Briefly, kidney sections were first incubated with 3% hydrogen peroxide (H_2_O_2_) solution for 10 min. The sections were washed thrice with PBS and then boiled in citrate antigen retrieval solution for 25 min in a microwave oven under high power. After cooling, the sections were incubated in blocking buffer for approximately 1 h to block nonspecific binding signals. Afterward, the sections were incubated with the corresponding primary antibodies (collagen-1, fibronectin, α-SMA, E-cadherin and Vimentin, Abcam) at 37 °C for 1 h. After the sections were washed thrice with PBS, the sections were incubated with a horseradish peroxidase (HRP)-labeled secondary antibody for an additional 1 h. Then, positive signals were developed with 3,3-diaminobenzidine (Sigma). Ten fields per slide were randomly selected for imaging. Positive signals were then quantified by Image Pro-Plus 6.0 software.

### Cell culture and treatments

Normal rat kidney (NRK)49F fibroblast cells were obtained from the American Type Culture Collection (Manassas, VA, USA) and cultured in Roswell Park Memorial Institute 1640 medium (Gibco, Grand Island, NY, USA) supplemented with 10% (v/v) FBS (GIBCO, Grand Island, NY, USA). Cells were seeded onto a 35-mm dish to reach confluence of 60%, then cells were serum-starved for 24 h. Subsequently, 5-ng/mL recombinant human TGF-β1 and (or) 10, 20 or 40-µM DIM were added in the medium to treat the cells. DIM was dissolved in dimethyl sulfoxide (DMSO, Sigma-Aldrich Company Ltd., Dorset, UK) and added to the medium 2 h before TGF-β1 treatment. The proliferation rate of the cells was evaluated via MTT assay (Sigma, Saint Louis, MO, USA) in accordance with the manufacturer’s protocol. Absorbance (A) was measured at 490 nm. Cell proliferation rate was calculated with the following formula: cell proliferation rate (%) = (A of the experimental group/A of the control group) × 100%.

### Immunofluorescence staining

NRK49F cells were seeded on coverslips and treated as previously described. After 24 h of treatment with recombinant human TGF-β1, the cells were fixed in 4% paraformaldehyde, permeabilized with 0.2% Triton X-100 (Boster Bioengineering Co., Ltd.) in PBS, and then blocked in 5% bovine serum albumin (BSA; Guge Biotechnology Co., Ltd.) for 30 min. Afterward, cells were cultured overnight with 1:100 dilutions of primary antibodies targeting vimentin and α-SMA. Then, cells were incubated for 1 h with 1:200 dilution of secondary antibody (Guge Bioengineering Co., Ltd., Wuhan, China). Lastly, the coverslips were mounted onto glass slides with an anti-fade reagent with DAPI (Beyotime Institute of Biotechnology). Immunofluorescence staining was quantified on coded cell coverslips as the integrated option density (IOD) value.

### Western blot analysis

Cell lysates were prepared in RIPA (Biyuntian, Haimen, China) lysis buffer with 1% cocktail (Biyuntian, Haimen, China). Protein concentrations were measured via the BCA method. Then cell lysate was added to each well and separated by SDS-polyacrylamide gel electrophoresis. Subsequently, the proteins were electrophoretically transferred to a polyvinylidene fluoride membrane (Millipore, Billerica, MA, USA). After blocking with 5% nonfat milk for 2 h, the membranes were incubated with specific primary antibodies including anti-p-Smad3, Smad3, p-Smad2, Smad2, Smad7 (Cell Signaling Technology, USA), and β-actin (Santa Cruz, USA) at 4 °C overnight. Afterward, the membranes were incubated with HRP-conjugated secondary antibody (LICOR, USA) for 60 min. Finally, the blots were scanned with a two-color infrared Li-Cor scanner (Odyssey, LICOR, USA).

### Statistical analyses

Data were expressed as mean ± SD. Results were statistically analyzed via one-way analysis followed by the Student–Newman–Keuls method (GraphPad Prism 5.0, San Diego, CA, USA). *p* < .05 was considered statistically significant.

## Results

### DIM ameliorates kidney injury in UUO mice

**Figure 1.**
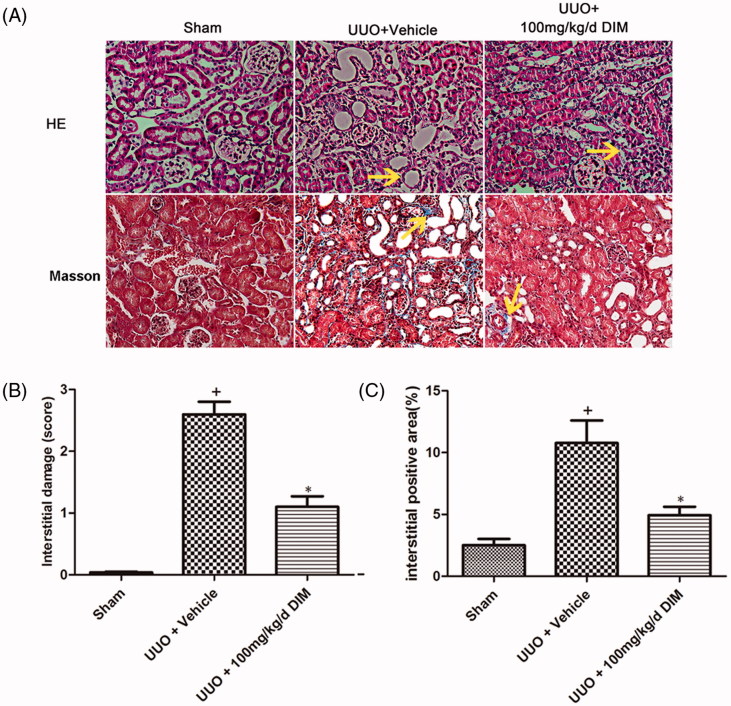
DIM ameliorating kidney injury in UUO mice. (A) Hematoxylin and eosin (HE) and Masson staining (200×) of three different groups, arrow, positive signals. (B) Quantitative analysis of interstitial damage score in three groups. (C) Quantitative analysis of interstitial collagens accumulation assessed by Masson staining, blue area. +*p* < .05 vs. the sham group and *p < .05 vs. UUO + the vehicle group.

To evaluate the protective effect of DIM on kidney injury, the UUO model was constructed. UUO model mice were treated with 100 mg/kg/day DIM. As shown in Figure 1(A), compared with the sham group mice, the UUO model mice exhibited extensive kidney structural damage and morphology alteration characterized by tubular epithelial atrophy, tubular lumen dilation, interstitial inflammatory cell infiltration, and tubular necrosis. Treatment with 100 mg/kg/day DIM significantly (*p* < .05) prevented interstitial injury at day 7 after UUO. The interstitial injury score in the DIM-treated group was lower than that in the UUO + vehicle-treated group as presented in Figure 1(B). Masson trichrome staining showed a lower degree of interstitial collagen deposition in the 50 mg·kg–1·day–1 CTS-treated group than in the vehicle-treated group (Figure 1(A,C)) on day 7 after UUO. These results suggested that DIM markedly ameliorated the histological features associated with renal tubular injuries after 7 days of UUO.

### DIM attenuates ECM deposition after UUO

**Figure 2.**
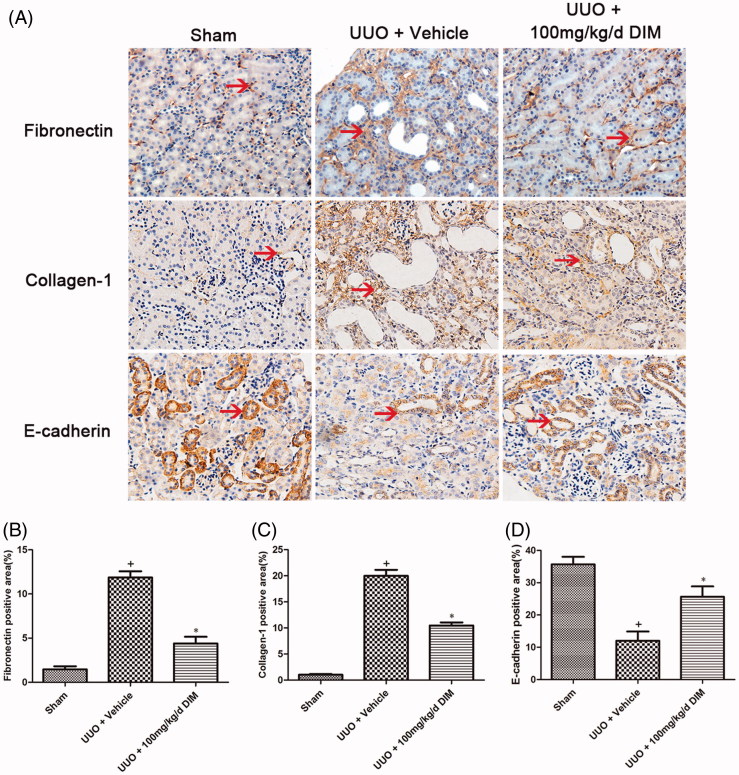
DIM attenuating ECM deposition in UUO mice. (A) Immunohistochemistry staining of fibronectin, collagen-1 and E-cadherin (magnification, 200×) in the three groups: (B), (C) and (D). Semi-quantitative immunohistochemistry staining of fibronectin, collagen-1 and E-cadherin in the three groups. + *p* < .05 vs. the sham group and *p < .05 vs. UUO + the vehicle group. Arrow, positive signal.

Fibronectin and collagen-1 are the most important components of accumulated ECM, and E-cadherin is the hallmark of the tubular cells. Both components dramatically increase in various models of chronic kidney injury. In Figure 2(A), immunohistochemistry staining revealed that fibronectin and collagen-1 expression levels were barely detected in the sham-operated group. However, their expression levels significantly increased after 7 days of UUO. Compared with the UUO group, the DIM group exhibited markedly decreased expression levels of fibronectin and collagen-1. Additionally, the quantitative immunohistochemical analysis of collagen-1 and fibronectin revealed that renal fibrogenesis markedly decreased in the UUO model after DIM treatment (Figure 2(B,C)). Furthermore, the expression of E-cadherin decreased in the obstructed kidneys, while the treatment of CTS reversed this effect.

### DIM inhibits UUO-induced myofibroblast activation

**Figure 3.**
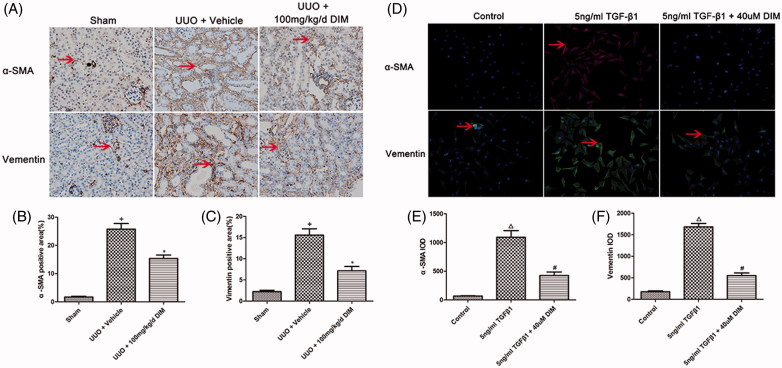
Analysis of fibroblast-to-myofibroblast transition *in vivo* and *in vitro*. (A) Immunohistochemistry staining of α-SMA and vimentin (magnification, 200×) in the sham and the UUO models; (B) and (C) Semi-quantitative immunohistochemistry staining of α-SMA and vimentin in the sham group and the UUO model; (D) Immunofluorescence of α-SMA and vimentin in TGF-β1-activated fibroblast cell model. (E) and (F) IOD value of α-SMA and vimentin in the cell model. +*p* < .05 vs. the sham group, *p < .05 vs. UUO + the vehicle group, Δp < .05 vs. the control group, #*p* < .05 vs. the 5ng/ml TGF-β1 group; Arrow, positive signal.

Myofibroblasts are the principal cells that produce ECM proteins during the progression of kidney fibrosis. Myofibroblast differentiation is marked by increased expression levels of α-SMA and vimentin. As shown in Figure 3(A), the 7-day UUO mice displayed increased α-SMA–positive cells in obstructed kidneys, whereas mice treated with 100 mg/kg/day DIM exhibited significantly (*p* < .05) decreased α-SMA-positive cells in obstructed kidneys. Immunohistochemistry staining was utilized to detect the expression of vimentin, another myofibroblast marker. UUO mice exhibited significantly (*p* < .05) increased vimentin expression in kidney interstitium, whereas DIM-treated mice showed a considerably lower expression of vimentin, as illustrated in Figure 3(A). The *in vitro* experiments showed similar results. Upon rhTGF-β1 treatment for 24 h, the NRK49F cells showed markedly increased expression levels of α-SMA and vimentin. These effects were preserved by treatment with 40-µM DIM. These results indicated that DIM inhibited the transition of renal fibroblasts to myofibroblast in renal fibrosis, as depicted in Figure 3(D–F).

### DIM protects against TGF-β1-induced fibroblast proliferation

**Figure 4.**
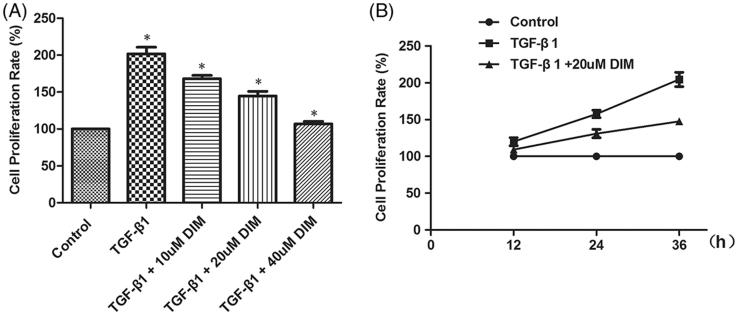
DIM inhibiting the TGF-β1-induced proliferation of NRK49F cells in a (A) dose- and (B) time-dependent manner. The proliferation rate of the NRK49F cells was measured via MTT assay. *p < .05 vs. the former group.

TGF-β1 could increase fibroblast proliferation in adult kidney fibrosis and activate the differentiation of fibroblasts into myofibroblasts, which are responsible for ECM production. The effect of DIM on the TGF-β1-induced proliferation of NRK49F cells was detected. After 36 h of TGF-β1 treatment, the proliferation rate of NRK49F cells markedly increased by 101.67%. DIM attenuated the TGF-β1-induced proliferation of the NRK49F cells in a dose-dependent manner (Figure 4(A)). Further analysis revealed that 20-µM DIM inhibited the TGF-β1-induced proliferation of NRK49F cells in a time-dependent manner (Figure 4(B)).

### TGF-β1/Smad signaling is involved in DIM-attenuated fibroblast inactivation

**Figure 5.**
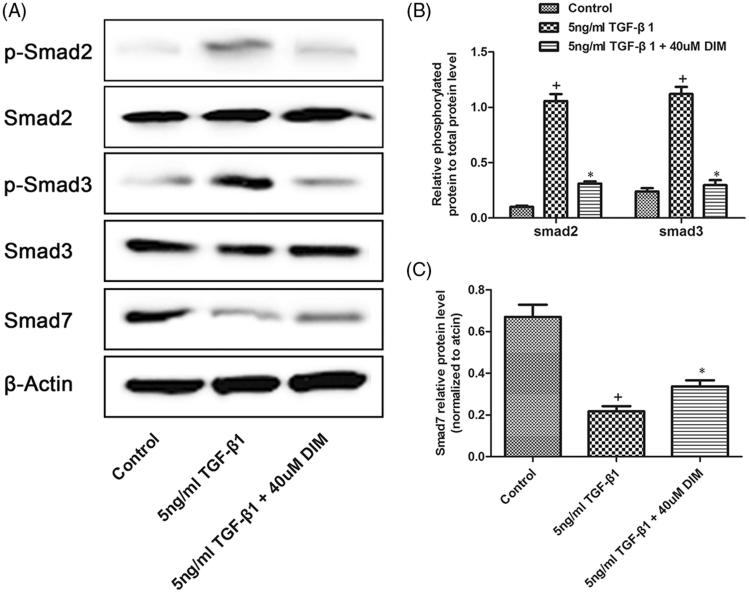
Smad-dependent TGF-β1 signaling pathways measured using Western blot. (A) NRK49F cells were treated with 5-ng/mL TGF-β1 and (or) 40-µM DIM for 2 h, and then Western blot was performed to detect Smad-dependent-related proteins. (B and C) Semi-quantitative analysis of the relative expression of different proteins after DIM treatment. +*p* < .05 vs. the control group and *p < .05 vs. the 5-ng/mL TGF-β1-treated group.

The TGF-β1/Smad signaling pathway is one of the most important pathways that induce myofibroblast activation. Smad2/3 is the primary effector of this signaling pathway, whereas Smad7 exerts an antagonistic effect. Therefore, we tested the influence of DIM on the activation of Smad proteins in the TGF-β1-induced activated NRK49F cell model. As shown in Figure 5(A), the phosphorylation levels of Smad2 and Smad3 increased with 2 h of TGF-β1 treatment, whereas that of Smad7 decreased. Interestingly, the phosphorylation of Smad2 and Smad3 was significantly inhibited in DIM-treated NRK49F cells. DIM treatment, however, did not affect the total protein levels of Smad2 and Smad3. Moreover, the high expression of Smad7was retained after DIM treatment. Collectively, our data indicated that DIM inhibits myofibroblast activation by regulating the TGF-β/Smads signaling pathway.

## Discussion

We first demonstrated that DIM significantly ameliorated kidney injury and renal interstitial fibrosis in a UUO mouse model. Additionally, DIM markedly decreased fibroblast activation in the UUO model, as confirmed by the decreased expression levels of the myofibroblast markers α-SMA and vimentin in DIM-treated mice. *In vitro* data indicated that DIM inhibited the TGF-β1-induced proliferation of NRK49F cells in a dose- and time-dependent manner. These observations showed that the mechanisms that underlie the inhibitory effect of DIM on fibroblast activation may be related to the regulation of the TGFβ1/Smads signal.

However, a variety of cell types have been reported to play important roles in the pathogenesis of kidney fibrosis. But until now, mounting evidence supports that fibroblasts are the principal source of myofibroblasts producing ECM in injured kidney. Producing cells, which acquire the phenotype of myofibroblast and generate a large amount of interstitial matrix upon activation, are the principal matrix producing cells [17,18]. Therefore, we choose renal fibroblast as a cell model to investigate the effect of DIM on renal fibrosis. We performed both *in vivo* and *in vitro* studies using UUO surgical mice and TGF-β1-treated renal fibroblasts, respectively. During renal fibrosis, fibroblasts generally initiated two processes: proliferation and ECM production, including fibronectin, collagen-1 and IV, heparin, laminin and perlecan. Our study confirmed resident fibroblast proliferated after the treatment of TGF-β1, with evidence of increased NRK49F cell proliferation rate tested by MTT assay. Furthermore, both *in vivo* and *in vitro* model showed fibroblasts undergo phenotype translation evidenced by high-expressed myofibroblasts markers like α-SMA and vimentin, as well as excessive production of ECM like fibronectin and collagen-1 in UUO model. Interestingly, our study found that DIM exerts significantly effects of anti-fibrosis in kidney, and fibroblasts phenotype translations were inhibited *in vivo* and *in vitro* after DIM treatment. In addition, the *in vitro* experiment showed that DIM inhibited local fibroblast proliferation, thus decreasing the sources of myofibroblasts. These data provided theoretical basis for its clinical study for the treatment of CKD patients in future.

The activation or inactivation of TGF-β/Smad signaling pathway, as reflected by the expression levels of Smad2, Smad3 and Smad7, is a pathogenic mechanism in the progression of renal fibrosis [19]. Previous studies have indicated that Smad3 is highly activated during fibrogenesis and could lead to the degradation of Smad7 via a ubiquitin–proteasome degradation pathway [20]. Meanwhile, the upregulation of Smad7 degrades type-I TGF-β receptors and thus inhibits the activation of the TGF-β/Smadsignal [21]. Therefore, the specific targeting of the TGF-β/Smad3 signaling pathway via Smad7 upregulation is a potentially effective therapeutic strategy for kidney fibrosis. Moreover, Smad2 and Smad3 are expressed at higher levels in patients with CKDs than in those with normal conditions. Smad3 is a fibrogenic mediator; the targeted disruption of TGF-β1/Smad3 signaling protects against renal interstitial fibrosis in obstructive nephropathy [22]. Notably, the inhibition of the TGF-β1-induced phosphorylation of Smad3 suppresses the proliferation of myofibroblasts and the differentiation of renal fibroblasts from myofibroblasts [23]. In contrast to Smad3, Smad2 cannot directly bind to the promoter of pro-fibrotic genes; Smad2 exerts a renal protective role by inhibiting Smad3 phosphorylation and binding with target genes [24]. This study demonstrated that DIM treatment significantly inhibited TGF-β/Smad signaling via inactivating Smad2/3 phosphorylation and increasing Smad7 expression in the TGF-β1-activated fibroblast model. These results indicated that the induction of Smad7, but not Smad2, restores the imbalance between the TGF-β/Smad signaling pathways, which may be a potential mechanism for the suppression of myofibroblast activation by DIM.

In conclusion, our findings identified DIM as a potential therapeutic agent in progressive renal interstitial fibrosis. The possible mechanism through which DIM attenuates renal interstitial fibrosis in the UUO mouse model is related to the inactivation of local fibroblasts through the induction of Smad7, thus suppressing TGF-β1-Smad2/3 signaling activation.
